# Successful treatment of pediatric IgG4 related systemic disease with mycophenolate mofetil: case report and a review of the pediatric autoimmune pancreatitis literature

**DOI:** 10.1186/1546-0096-9-1

**Published:** 2011-01-04

**Authors:** Melissa Mannion, Randy Q Cron

**Affiliations:** 1 Department of Pediatrics, University of Alabama at Birmingham School of Medicine, Birmingham, AL, USA; 2 Division of Rheumatology, Children's Hospital of Alabama, Birmingham, AL, USA

## Abstract

Autoimmune pancreatitis is frequently associated with elevated serum and tissue IgG4 levels in the adult population, but there are few reports of pediatric autoimmune pancreatitis, and even fewer reports of IgG4 related systemic disease in a pediatric population. The standard of care treatment in adults is systemic corticosteroids with resolution of symptoms in most cases; however, multiple courses of corticosteroids are occasionally required and some patients require long term corticosteroids. In these instances, steroid sparing disease modify treatments are in demand. We describe a 13-year-old girl with IgG4 related systemic disease who presented with chronic recurrent autoimmune pancreatitis resulting in surgical intervention for obstructive hyperbilirubinemia and chronic corticosteroid treatment. In addition, she developed fibrosing medianstinitis as part of her IgG4 related systemic disease. She was eventually successfully treated with mycophenolate mofetil allowing for discontinuation of corticosteroids. This is the first reported use of mycophenolate mofetil for IgG4 related pancreatitis. Although autoimmune pancreatitis as part of IgG4 related systemic disease is rarely reported in pediatrics, autoimmune pancreatitis is also characterized as idiopathic fibrosing pancreatitis. All pediatric autoimmune pancreatitis cases reported in the world medical literature were identified via a PUBMED search and are reviewed herein. Twelve reports of pediatric autoimmune pancreatitis were identified, most of which were treated with corticosteroids or surgical approaches. Most case reports failed to report IgG4 levels, so it remains unclear how commonly IgG4 related autoimmune pancreatitis occurs during childhood. Increased evaluation of IgG4 levels in patients with autoimmune pancreatitis may shed further light on the association of IgG4 with pancreatitis and the underlying pathophysiology.

## Background

The concept of immune mediated pancreatitis was first suggested by Sarles et al. in 1961 after observing massive lymphoplasmocytic infiltrates and fibrosis on pancreatic histology in patients with pancreatitis of unknown etiology [1]. Autoimmune pancreatitis (AIP) was further documented and characterized by Yoshida et al. in 1995 [2] with 11 common characteristics that have become the basis for diagnostic criteria of AIP [3-5] (Tables 1 and 2). These criteria are based on characteristic changes seen on imaging, pathology, serology, and disease modification (and usually resolution) with corticosteroid treatment [2-5]. When Hamano et al. identified an abnormal fraction of gamma globulins in patients with AIP in 2001, elevated serum IgG4 level became the hallmark lab value for AIP in adults [6]. Since then there have been many reports of adults with isolated AIP and multisystem sclerosis with elevated IgG4 levels [7]. However, there have been very few reports of pediatric patients with AIP [8-13], and all of those have had normal or not reported IgG4 levels. There is one report of a pediatric patient with elevated IgG4 levels; however, she did not have imaging changes or histology available to make a more definitive diagnosis of AIP [14]. Herein, an adolescent case of AIP [by Japanese Pancreas Society (JPS) 2006 criteria [4] (Table 1) and Mayo Clinic HISORt criteria [3,5] (Table 2)] with elevated IgG4 levels and evidence of systemic disease treated successfully with mycophenolate mofetil (MMF) is reported. A review of the literature on the topic of IgG4 related systemic disease and AIP in childhood is included.

**Table 1:** Clinical diagnostic criteria of Autoimmune Pancreatitis as proposed by the Japanese Pancreas Society, revised 2006 (4)

1.	Diffuse or segmental narrowing of the main pancreatic duct with irregular wall and diffuse or localized enlargement of the pancreas by imaging studies, such as abdominal ultrasonography, computed tomography, and magnetic resonance imaging.
2.	High serum gamma-globulin, IgG, or IgG4, or the presence of autoantibodies, such as antinuclear antibodies and rheumatoid factor.

3.	Marked interlobular fibrosis and prominent infiltration of lymphocytes and plasma cells in the periductal area, occasionally with lymphoid follicles in the pancreas.

**Table 2:** Mayo Clinic diagnostic groups with HISORt criteria (3,5)

Group A:	Diagnostic pancreatic histology (presence of 1 or both of the following): Resection specimen or core biopsy showing the full spectrum of changes of LPSP, and/or ≥ 10 IgG4 positive cells/hpf; IgG4 on immunostain of pancreatic lymphoplasmacytic infiltrate.
Group B:	Typical imaging + serology (presence of all of the following criteria): CT or MRI showing diffusely enlarged pancreas with delayed and "rim" enhancement; pancreatogram showing diffusely irregular pancreatic duct; elevated serum IgG4 levels.

Group C:	Response to steroids (presence of all of the following criteria): Unexplained pancreatic disease after negative work-up for known etiologies including cancer; elevated serum IgG4 levels and/or other organ involvement confirmed by presence of abundant IgG4-positive cells; resolution/marked improved in pancreatic and/or extrapancreatic manifestations with steroid therapy.

## Case Presentation

A 13-year-old Caucasian female with chronic abdominal pain initially developed fever, headache, and joint symptoms. Her symptoms persisted and progressed until she was hospitalized for shortness of breath several months later after developing what appeared to be a right lung scar and pericardial effusion by imaging. She was also diagnosed with pancreatitis and jaundice with a serum bilirubin level of 4.5 mg/dL. A computerized tomography (CT) scan revealed multiple pancreatic masses and biliary dilatation, lab work revealed an elevated IgG4, and she was transferred to our institution for further work up and management. Because of her persistent and recurrent pancreatitis symptoms, along with abnormal imaging she was diagnosed with AIP, meeting both JPS criteria (criterion 1 and criterion 2) and HISORt criteria (group B and after initiation of steroids, group C). Radiographic imaging [endoscopic ultrasound (EUS) and endoscopic retrograde cholangiopancreatography (ERCP)] and surgical placement of a pancreatic stent were performed. Prior to stent placement her serum lipase was elevated at 313 U/L (10-180). Other significant laboratory findings included the following results: amylase 39 U/L (39-100), AST 106 U/L (10-30), ALT 88 U/L (10-30), and alkaline phosphatase 417 U/L (93-386). EUS/ERCP revealed a stricture at the distal common bile duct, multiple focal hyperechoic areas in the pancreas with benign fine needle aspirate (FNA) consistent with chronic pancreatitis (specifically revealing a lymphocytic inflammatory infiltrate; no staining was performed on pancreatic aspiration specimens because there was insufficient sample), and a pancreatogram consistent with chronic pancreatitis (stricture of distal common bile duct and biliary dilatation). Following stent placement her lipase improved to 84 U/L. The stent was removed two months later as previously scheduled, and shortly after the procedure she developed worsening abdominal pain. A CT of the abdomen demonstrated dilatation of the intrahepatic and extrahepatic bile ducts without evidence of choledocholithiasis, mild dilation of the pancreatic duct and multiple foci of hypodensity within the kidneys. Her bilirubin level and liver function tests began to increase. One week after stent removal she was taken back for an ERCP and another stent was replaced. Her pain and jaundice improved, and the liver enzymes quickly returned to normal. When her stent was removed for the second time, multiple brushings and biopsies were taken. Pathology on duodenal biopsies revealed marked expansion of atypical lymphoid tissue, positive staining for CD20, BCL-6, BCL-2, CD10, and KI-67 and negative for CD5, CD23, Tdt, BCL-1, and CD30. cMYC translocation was negative and there was a light chain but not heavy chain rearrangement present. IgG4 staining was not done on this sample. This immunochemistry suggested a diffuse large B cell lymphoma; however, follow up on following FNA, biopsy and imaging did not demonstrate a monoclonal lymphoid population nor suggest malignancy. Autoantibody studies were all negative including: ANA, anti-dsDNA, anti-smooth muscle antibodies, anti-RNP, SSA/SSB autoantibodies, scleroderma-70 antibodies, and ANCA. In addition, C3, C4, and angiotensin converting enzyme levels were within normal limits for age. She returned again in 3 months for stent removal which she tolerated well. EUS revealed calcifications in the head of the pancreas (a finding that is unusual for autoimmune pancreatitis) and a large (27 mm × 16 mm) area of stranding within the mediastinum, consistent with fibrosing mediastinitis. As treatment for her pancreatitis, corticosteroids had been prescribed previously resulting in improvement of pain but with significant weight gain, and her abdominal pain/symptoms would return shortly after tapering the corticosteroid dose. At that time the rheumatology service was consulted for the possibility of AIP.

To spare corticosteroids and to treat the IgG4-related pancreatitis and mediastinitis, she was started on MMF and tapered up to a maximum dose of 1,000 mg twice daily. She improved quickly and remarkably with resolution of abdominal pain and the ability to return to an enteral diet within days of starting MMF. The addition of MMF allowed for significant improvement of symptoms, eventually in the absence of corticosteroids, as well as normalization of laboratory and imaging findings consistent with pancreatitis. In terms of her laboratory findings, her lipase improved from 444 to 53 U/L (normal, 10-180) and amylase lowered from 63 to 41 U/L within 3 days of MMF. The initial IgG4 level was 226 mg/dL (normal range, 11-157) prior to MMF; after 15 months of initiating treatment with MMF, the IgG4 subclass level was 73.4 mg/dL. Inflammatory markers, ESR and CRP, were not measured until after treatment was started, at which point they were within normal limits. A repeat chest and abdominal CT one year after initiating treatment did not reveal any residual mediastinal or hilar lymphadenopathy. There was no evidence of pulmonary nodules or focal pancreatic lesions, and there was resolution of the hypodense renal lesions and fatty infiltration of the liver. She was ultimately tapered off of all immunosuppressive therapy within 1 year of treatment with MMF. The child currently remains in good health off of all immunosuppressive medicine after an additional period of over 7 months. She, thus, represents the first pediatric patient with AIP and elevated IgG4 levels to be successfully treated with MMF allowing for discontinuation of corticosteroids, and eventually all immunosuppressive therapy.

### Review of the AIP literature and focus on pediatric cases

Pancreatitis due to an inflammatory, autoimmune process was first suggested by Sarles et al. in 1961 with the description of 10 adult cases of chronic pancreatits, all with massive cellular infiltrates and increased inflammation [1]. AIP was later named and described by Yoshida et al. [2] in 1995 when a patient without evidence of Sjogren syndrome developed pancreatitis and responded to corticosteroid therapy. The patient had evidence of autoimmune abnormality with a positive ANA titer, anti-thyroglobulin antibody, and anti-microsomal antibodies; all other autoantibodies tested were negative [2]. The authors described 11 characteristics of AIP that have been used as the basis for the development of recent diagnostic criteria including; the JPS diagnostic criteria of AIP [4] and the Mayo Clinic HISORt criteria [3,5] (Table 1).

### Diagnostic criteria for AIP

Diagnostic criteria for AIP were first developed by the JPS in 2002 and were revised in 2006 after reevaluating additional patients with AIP and with likely AIP. The foundation of the diagnosis rests on meeting characteristic findings on imaging, along with supportive serology and/or histology (Table 1). The JPS criteria emphasize the importance of excluding malignancy during the work up and the responsiveness to steroids, although they did not include it as a diagnostic criterion [4]. The Mayo Clinic, Chari et al. [3,5], used a cohort of patients with AIP diagnosed by histology to develop criteria in five categories that make 3 diagnostic groups (Table 2). Those categories and criteria are: Histology, [at least one of the following: periductal lymphoplasmacytic infiltrate with obliterative phlebitis and storiform fibrosis (LPSP) or lymphoplasmacytic infiltrate with storiform fibrosis showing abundant (≥10 cells/hpf) IgG4-positive cells]; Imaging, typically diffusely enlarged gland with delayed (rim) enhancement, diffusely irregular, attenuated main pancreatic duct or alternatively, focal pancreatic mass/enlargement, focal pancreatic duct stricture, pancreatic atrophy, pancreatic calcification, or pancreatitis; Serology, elevated serum IgG4 level (>140 mg/dL); Other organ involvement, hilar/intrahepatic biliary strictures, persistent distal biliary stricture, parotid/lacrimal gland involvement, mediastinal lymphadenopathy, retroperitoneal fibrosis; and response to steroid therapy, resolution/marked improvement of pancreatic/extrapancreatic manifestations with steroid therapy [3,5]. Of the previously diagnosed idiopathic fibrosing pancreatitis (IFP) pediatric patients included in this review, by imaging and histology, they are more consistent with AIP, although immunoglobulin levels were not measured and steroids were not given (surgically treated) in any of the cases [15-18] (Table 3). Thus, for many pediatric patients reported in the literature to have IFP, it is difficult to establish whether they meet AIP criteria.

**Table 3:** Clinical characteristics of pediatric patients diagnosed with AIP and IFP (3-5).

	Case report	Patient and demo-graphics	Signs/ symptoms	Histology	Imaging	Serology (mg/dl)	Other organ systems	Response to steroids	Treatment	Outcome
AIP/likely AIP	this report	13yo female	fever, headache, joint pain, vomiting, epigastric pain, shortness of breath, weight loss, jaundice	EUS-FNA consistent with chronic pancreatitis, duodenal ampulla enlarged, duodenal biopsy with atypical lymphocytic infiltrate	focal hypoechoic areas in head, body, and tail of pancreas with enlargement of the head of the pancreas, distal stricture of CBD with biliary dilatation	IgG4 226, other autoantibodies negative	mediastinal fibrosis, pulmonary nodules, multiple hypodense foci in kidneys,	improvement in pain, unable to taper without return of symptoms	mycophenolate mofetil	resolution of symptoms and abnormalities on imaging, normalization of IgG4

	Fukumori et al. (14)	17yo female	severe epigastric and back pain	not done	US, CT normal, MRCP-MPD only in head of pancreas	IgG 2155, IgG4 157, positive antilactoferrin Ab, ANA 1:80 (speckled), other autoantibodies negative	none noted	resolution of pain, entire MPD visualized on MRCP, disappearance of ALF Ab	30 mg prednisolone, tapered off at 8 months	asymptomaticand not currently treated

	Pace et al. (8)	18yo male	recurrent acute pancreatitis and cholestasis	lymphocytes, macrophages, plasma cells consistent with AIP	enlarged pancreatic head on endoscopy	IgG4 23, ANA 1:320, other autoantibodies negative	none reported	resolution of symptoms	initially started on prednisolone then tapered off	asymptomatic without further treatment

	Blejter et al. (9)	16yo male	pruritus and weight loss	chronic pancreatitis with interstitial periductal lymphoplasmacytic infiltration and interstitial fibrosis	enlarged pancreatic head, dilated biliary tract, no passage of contrast into duodenum on cholangio-graphy	IgG4 normal, hypogammaglob-ulinemic, (no specific values given), other autoantibodies negative	none reported	resolution of symptoms, repeat cholangiogram without biliary dilatation or stricture	prednisone 40 mg/kg/day then tapered	no recurrence, he requires NPH insulin for diabetes

	Refaat et al. (10)	11yo male	nausea, vomiting, dull epigastric pain, anorexia, diarrhea	periductal fibrosis, lymphocyte-plasmic parenchymal infiltrate	enlarged hypoechoic pancreatic head (US), hypointense surrounding rim (MRI T2-W), diffuse irregular narrowing of main pancreatic duct	IgG4 and IgG normal, (no specific values given), other autoantibodies negative	none reported	not reported	not reported	not reported

	Gargouri et al. (11)	10yo male	severe abdominal pain, biliary vomiting, weight loss	not done	enlarged pancreas (US), multiple stenoses of Wirsung duct (MRCP), multiple stenoses without intracanalar lacuna and stenosis of retropancrea-tic segment of the bile duct (ERCP)	IgG and IgG4 normal (no specific values given), autoantibodies negative	none reported	resolution of symptoms, normalization of pancreatic size and stenoses of Wirsung duct	IV steroids 1 mg/kg/day × 10 days then decreased and discontinued at 7 months	asymptomatic, reported 4 years after discontinuation of steroids

	Takase et al. (12)	14yo female	severe right upper quadrant pain	not done	enlargement of pancreas head to tail, homogeneous low-echoic area with some high-echoic spots inside (US), enlargement of the head of the pancreas (CT, MRI), enlarged main pancreatic duct with narrow distal portion (MRCP)	IgG 2104 (high) IgG4 54, other autoantibodies negative	none reported	improvement in symptoms, IgG, MRCP, relapse × 2 (minimum)	initial dose IV prednisolone 30 mg/day, multiple tapers and steroid burst, required daily treatment	multiple relapses with subsequent imaging changes, no return of elevated IgG

	Bartholomew et al. (13)	10yo male	jaundice, intermittent abdominal pain, fatigue, weight loss	chronic pancreatitis secondary to lymphoplasmacytic sclerosing pancreatitis	pancreatic head mass with likely invasion of portal and superior mesenteric veins (EUS)	not reported	none reported	not given	Whipple pancreatico-duodenectomy	symptom free at 6 month follow up, requires digestive enzymes

**Diagnosed as IFP**, examples of case reports that could be consistent with AIP	Atkinson et al. (15)	10yo male	epigastric pain, jaundice	fibrosis enclosing normal acini, with lymphocytes, plasma cells, and leukocytes between acini	mass in pancreatic head (laparotomy), obstruction of CBD (IOC)	not reported	none reported	not given	cholecysto-duodenostomy	symptom free 15 years following surgery

	Elitsur et al. (16)	2yo female	abdominal pain	fibrous replacement of pancreatic tissue, preservation of Islets of Langerhans, with polymorpho-nuclear cells and plasmalymph-ocytic cells	enlarged pancreas with dilatation of proximal bile duct (US, CT), enlarged nodular pancreas with suggestion of retroperitoneal mass (MRI)	ANA, anti smooth muscle, anti mitochondrial, antithyroid antibodies all negative, immunoglobulins not reported	retroperitoneal mass	not given	ex-lap for diagnosis, spontaneous resolution of obstruction	repeat US revealed normal pancreas and CBD

	Stephen et al. (17)	7yo male	jaundice, lethargy, weight loss, prior to jaundice abdominal cramping and vomiting	nodular aggregates of lymphocytes and plasma cells, acinar tissue replaced by dense connective tissue	enlarged pancreas, head less echogenic than the rest of the pancreas, dilated CBD with tapering at the head of pancreas (US)	negative ANA, no other studies reported	cholangitis, pericholangitis (similar inflammatory infiltrate)	not given	Roux-en-Y cholecysto-jejunostomy, incidental appendectomy	symptom free

	Keil et al. (18)	14yo male	epigastric pain, jaundice	severe fibrosis with chronic lymphocytic inflammatory infiltrate	enlarged edematous head of pancreas, dilated CBD (US, CT), 2 cm stenosis of CBD (ERCP)	"biochemical parameters of inflammatory reactions were normal"	none reported	not given	biliary stenting	normal pancreas by US after 12 months of stenting, symptom free 3.5 years following treatment

Abbreviations used: AIP - autoimmune pancreatitis, EUS - endoscopic ultrasound, FNA - fine needle aspirate, CBD - common bile duct, US - ultrasound, CT - computed tomography, MRCP - magnetic resonance cholangiopancreatography,

MPD - main pancreatic duct, Ab - antibody, ANA - antinuclear antibody, ALF - antilactoferrin antibody, MRI - magnetic resonance imaging, T2-W - T2 weighted images, ERCP - endoscopic retrograde cholangiopancreatography, IOC - intraoperative cholangiogram

### Demographic and clinical characteristics of AIP

Typically, patients with AIP are older males; in one study, 79% of patients were more than 50 years old (age range 14-85 years) and 83% were male [3], and in another study, the age range was 38-73 years old and 75% were male [6]. The patients usually presented with obstructive jaundice [73% [3] and 65% [19]], weight loss (35%) [19], and non-specific abdominal pain [35% [19] and 34.5% [3]] that was mild and did not require narcotics [3]. In the pediatric case reports with a diagnosis of AIP by JPS or HISORt criteria, the ages ranged from 10-18 years old (above 18 years was considered an adult patient), and 62.5% were male [8-14] (Table 3). If the IFP cases included in this review are considered AIP, the pediatric age range becomes 2-18 years old and 66.7% of the patients were male [8-18]. Clinically these patients (both AIP and IFP) presented with varying degrees of abdominal pain (10/12 patients, 83%), weight loss (5/12 patients, 42%), and jaundice (5/12 patients, 42%) [8-18]. Abdominal pain is more frequently a presenting symptom of AIP in pediatric patients, like the one reported herein, than in adult patients. In addition to clinical criteria, histopathologic criteria have been developed to diagnose AIP.

### Histopathology of AIP

Similar to the patient reported herein, needle biopsy of the pancreas in the case report by Yoshida et al. [2] revealed severe fibrotic change with lymphocytic infiltration. LPSP was the descriptive name of AIP prior to the development of diagnostic criteria, a histologic pattern distinct from ordinary chronic pancreatitis, alcoholic pancreatitis, and obstructive pancreatitis secondary to cancer. The histologic findings of AIP used to include or exclude a diagnosis of AIP in case series and cohort studies are a periductal lymphoplasmacytic infiltrate with obliterative phlebitis and swirling fibrosis centered around ducts and veins [3-5,19-25]. The majority of lymphocytes are T cells, and eosinophils and neutrophils can be found. In one study, all 3 patients with ulcerative colitis were found to have neutrophilic infiltration of pancreatic ducts [21]. Two subtypes of histologic inflammatory pattern were described, ductocentric and lobulocentric [21]. A similar dichotomy was described by another group, interlobular lymphoplasmacytic inflammation and idiopathic duct-centric chronic pancreatitis (intralobular) [26]. Thus, the histopathologic findings in AIP are rather varied.

### Imaging findings in AIP

Both the JPS [4] and the Mayo HISORt [3,5] criteria have specific imaging descriptions for AIP. A diffusely enlarged pancreas on CT with diffuse irregular narrowing of the pancreatic duct (both of which resolved following therapy) were included in the Yoshida et al. original description of AIP [2]. In fact, diffuse irregular narrowing of the pancreatic duct was part of the subjective differentiation between sclerosing pancreatitis with elevated IgG4 levels and ordinary chronic pancreatitis (irregular dilatation of the pancreatic duct or diffuse calcification) for Hamano et al. [6]. Typically, AIP will have imaging that reveals a "sausage-like" pancreas with delayed enhancement on CT scan, rim enhancement on T2 weighted MRI, or hypoechoic pancreas with echogenic spots on ultraound. AIP rarely causes calcification or pancreatic pseudocyst, but can cause localized enlargement which can contribute to the difficulty differentiating it from malignancy [7]. The JPS criteria for diagnosis of AIP require that pancreatic imaging is consistent with AIP [4]. The Mayo Clinic HISORt criteria, however, have 3 distinct diagnostic groups, only one of which includes imaging criteria [3,5]. Both criteria, though, have the same imaging requirement, an enlarged pancreas with diffuse irregular narrowing of the pancreatic duct [3-5].

### Elevated IgG4 levels in AIP

In addition to the imaging and histologic characteristics of AIP, a sensitive and specific serologic association with elevated IgG4 serum levels was first described by Hamano et al. [6]. A serum IgG4 level of at least 135 mg/dL resulted in 95% sensitivity and 97% specificity in differentiating between AIP (referred to as sclerosing pancreatitis in Hamano et al., and diagnosed by imaging, histology, and response to steroids) and pancreatic cancer. They did not find elevated serum IgG4 levels in patients with chronic pancreatitis, primary biliary cirrhosis, primary sclerosing cholangitis, nor Sjogren syndrome [6]. These findings were supported by investigators at the Mayo Clinic; using a serum IgG4 level cutoff of 140 mg/dL, the sensitivity for AIP was 76% and specificity 93%. Moreover, the negative predictive value of serum IgG4 <140 mg/dL was 99%, but the positive predictive value was only 36% [20]. In both the Mayo Clinic cohort [20] and the Hamano et al. cohort [6], 3-10% of patients without AIP had elevations in serum IgG4, and in the Mayo Clinic cohort 11/45 (24%) patients with AIP had normal serum IgG4 levels. All patients diagnosed with AIP regardless of serum IgG4 levels had similar clinical responses to corticosteroids. The patients most likely to have normal IgG4 levels with a histologic diagnosis of AIP were more likely to be female, younger, and less likely to have other organ involvement. In addition, all patients in the Mayo Clinic cohort that had normal serum IgG4 levels and available histology had positive IgG4 immunostaining [20].

IgG4 immunostaining has become a method to both confirm pancreatic histology consistent with AIP and evaluate other manifestations of a similar disease process. In the study by Ghazale et al. [20], 19/20 patients who had IgG4 immunostaining of pancreatic tissue showed >10 IgG4 positive cells/hpf. Five patients with elevated serum IgG4 levels that did not have AIP by histology, had IgG4 immunostaining performed that was negative in four patients and showed nonspecific focal clusters of IgG4 positive cells in one patient [20]. In another cohort, all 21 AIP patients with pancreatic tissue IgG4 immunostaining had at least >5 IgG4 positive cells/hpf, and extrapancreatic specimens with lymphoplasmacytic infiltrate (3 biliary, 2 pulmonary, 3 salivary glands, 4 renal) when tested also showed significant IgG4 positive cells by immunostaining (3 biliary, 1 lung, 1 salivary gland, 4 renal) [21]. Since the pathology of the extrapancreatic lesions is the same as the pancreatic pathology, the concept of IgG4 related systemic disease was developed. Whether or not distinct categories of AIP based on IgG4 levels will be established in pediatric cases is yet to be determined.

### Extrapancreatic manifestations of IgG4 related disease

Elevated serum IgG4 levels and positive IgG4 immunostaining have been associated not only with AIP, but also with retroperitoneal fibrosis, sialoadenitis, mediastinal adenopathy (as in the patient reported herein), cholangitis, and tubulointerstitial nephritis [3,21,22,27] and is one criterion for diagnosis in the HISORt criteria [3,5]. In 12 patients clinically diagnosed with idiopathic retroperitoneal fibrosis, 16 biopsies (retroperitoneal tissue, liver, kidney, colon, omentum) showed fibrosis with a lymphoplasmacytic infiltrate, and 14/16 biopsies had IgG4 positive plasma cells (not seen in one liver and one colon biopsy). Comparative biopsies from inflammatory and fibrotic diseases in similar organs failed to show any IgG4 positive cells [27]. Ghazale et al. [22] reported 4 patients with IgG4 positive immunostaining on bile duct resection with histology comparative to AIP, namely, lymphoplasmacytic infiltrate with storiform fibrosis and obliterative phlebitis around the bile duct, who had no evidence of pancreatic disease or involvement. Similarly, these patients responded to corticosteroids with improvement in all and resolution of strictures in most. Some patients, however, had relapse with corticosteroid taper, but instead of continuing low dose therapy they were started on other immunomodulatory medications, azathioprine, MMF, or cyclophosphamide with successful maintenance of treatment [22]. The similar pathology, immunostaining, and response to steroids have led to the proposal of an IgG4-related sclerosing disease, of which AIP is the pancreatic manifestation of a systemic process [7,21,23,27].

### Treatment of AIP

Prior to the concept of AIP, patients with painless swelling of the pancreas were treated with surgery to rule out a malignant process. The histologic specimens collected following resection, where malignancy was ruled out and an inflammatory infiltrate was noted instead, have been used to create the Mayo Clinic HISORt criteria [3]. Yoshida et al. [2] was the first to report the successful use of corticosteroids in a patient with AIP, resulting in resolution of symptoms, imaging abnormalities, and laboratory abnormalities. A resolution or at least effective response to corticosteroids is part of the Mayo Clinic HISORt diagnostic criteria and is noted as a characteristic of AIP in the JPS criteria [3-5].

As the concept of AIP as part of a systemic IgG4 related disease developed, so did the need for more aggressive treatment. In most cases of AIP, patients respond fully to a single course of corticosteroids [2,3,25]; however, some patients with AIP, and especially those with extrapancreatic manifestations (including our patient), are unable to fully wean off of corticosteroids [22,25,27]. Azathioprine and low dose prednisone have been used to control symptoms and limit disease course with success [27]; in addition, MMF and cyclophosphamide have also been used in cases of IgG4 associated cholangitis (IAC) with successful maintenance of remission [22]. MMF has also been used successfully in patients with idiopathic retroperitoneal fibrosis (there was no mention of IgG4 testing or staining in this case series) [28]. These patients were initially started on prednisone and MMF, the prednisone was weaned off over 6 months, and MMF was discontinued after 11-96 months (mean 27 months). All 9 patients had regression of disease and improvement in inflammatory markers; there was no recurrence after discontinuation of medical therapy. *In vitro *lab work has demonstrated that MMF has direct antifibrotic properties by inhibiting type I collagen production, altering the contractile and migratory functions of fibroblasts, and enhancing the production of matrix metalloproteinase (interstitial collagenase) [29]. Thus, MMF seems a logical choice in treating autoimmune fibrosing disorders.

### Pediatric Reports

There are 7 other pediatric patients reported in the literature with AIP or likely AIP (Table 3) by JPS or HISORt criteria. Only one had normal imaging [14] (she was unable to be diagnosed as AIP by JPS or HISORt criteria, but did respond to steroid therapy and had an elevated IgG4), and 4 of the 7 patients had histology reported, all 4 consistent with AIP [8-10,13]. The only patient with an elevated IgG4 was the patient who did not meet diagnostic criteria for AIP [14]; she also had an elevated total IgG level. One other patient had an elevated IgG level, but normal IgG4 level [12], and one patient did not have any laboratory work reported [13]. The other pediatric patients reported in the literature with AIP or likely AIP (Table 3) were treated with corticosteroids or pancreatoduodenectomy with mixed success. One patient requires daily steroids [12], treatment is not reported in one [10], the surgical patient requires digestive enzymes [13], and one patient requires NPH insulin for diabetes but did not have recurrence of disease [9]. There are many reports of IFP in the literature, but only 4 have either enough criteria information reported or criteria consistent with AIP (Table 3) [15-18]. These patients were treated with surgical resection [15,17], observation (following diagnostic exploratory laparotomy) [16], or biliary stenting [18], all with resolution of symptoms. None of these patients had immunoglobulins reported or were given corticosteroids. The extent of AIP and IgG4-associated autoimmunity in children remains unanswered at present.

## Conclusions

The patient presented in this report is diagnosable with AIP by both JPS and HISORt (group B or C) diagnostic criteria [3-5]. She also had an elevated IgG4 with evidence of extrapancreatic manifestations, consistent with IgG4 related systemic disease (ISD), even though IgG4 staining was not done (Table 1 and 2). Her symptoms improved with corticosteroids, but she was unable to wean off of them without disease flare and did not want to continue due to systemic side effects. She was successfully treated with MMF for one year with resolution of symptoms and evidence of inactive disease by imaging and laboratory studies for a total of 19 months, until she was lost to follow up. Our patient represents the first pediatric patient with elevated IgG4 who met both JPS and HISORt criteria for AIP, and the first pediatric patient with AIP/ISD who was successfully treated with MMF.

The pediatric patients reported in the literature, including the patient reported here, have a disease that is similar to adult AIP/ISD. The imaging reports are similar, histology (when available) is consistent with AIP, and there is typically a good response to corticosteroids. The pediatric patients, however, more often present with abdominal pain than the adult patients (11/12 reported here), and when immunoglobulins are measured, they are more likely to be normal than elevated. Our patient has criteria most similar to the adult patients and criteria reported in the literature, including an elevated IgG4 level which is only reported in one other pediatric patient, a patient that did not meet diagnostic criteria for AIP [14]. This current report demonstrates that while pediatric AIP/ISD is very similar to adult AIP/ISD, it is not identical. As reports of pediatric AIP/ISD increase, the criteria used to diagnose adults may need to be modified to better suit pediatric patients. Since pancreatic malignancy is significantly rarer in pediatric patients than adult patients, the criteria could likely be more inclusive without the risk of delaying treatment for cancer. If AIP/ISD were a more likely diagnosis in pediatric patients with pancreatitis, corticosteroid therapy could be attempted sooner and limit surgical interventions, symptoms, and hopefully disease progression. In the patients who do meet the adult criteria for AIP/ISD who are unable to wean off of corticosteroids or who cannot tolerate the side effects, other immunomodulatory medications should be considered. Our patient responded fully to 1 year of treatment with MMF, was symptom free at 19 months follow up with normal imaging and IgG4 levels.

## Consent

Institutional Review Board approval was attained. The patient and her family have been lost to contact. Repeated attempts at contact, including via her gastroenterologist, have failed.

## List of abbreviations

AIP: autoimmune pancreatitis; ISD: IgG4 related systemic disease; IFP: Idiopathic fibrosing pancreatitis; CT: computerized tomography; ERCP: endoscopic retrograde cholangiopancreatograhy; EUS: endoscopic ultrasound; FNA: fine needle aspirate; MMF: mycophenolate mofetil.

## Competing interests

The authors declare that they have no competing interests.

## Authors' contributions

MM conducted the literature search and data abstraction from the clinical chart, and was the primary author of the manuscript. RQC was involved in the patient care and participated in the manuscript's design and coordination and helped to draft the manuscript. Both authors read and approved the final manuscript.
